# Association of Serum Manganese Levels with Alzheimer’s Disease and Mild Cognitive Impairment: A Systematic Review and Meta-Analysis

**DOI:** 10.3390/nu9030231

**Published:** 2017-03-03

**Authors:** Ke Du, Mingyan Liu, Yanzhu Pan, Xin Zhong, Minjie Wei

**Affiliations:** 1 School of Pharmacy, Department of Pharmacology, China Medical University, Shenyang 110122, China; kdu@cmu.edu (K.D.); saffer@163.com (M.L.); Pyanzhu@126.com (Y.P.); zhongxin1004@sina.com (X.Z.); 2 Liaoning Key Laboratory of Molecular Targeted Anti-Tumor Drug Development and Evaluation, Shenyang 110122, China

**Keywords:** Alzheimer’s disease, mild cognitive impairment, manganese, serum, meta-analysis

## Abstract

Manganese (Mn) is one of the most studied environmental heavy metals linked to Alzheimer’s disease (AD). However, it remains unclear whether serum Mn levels are associated with AD and mild cognition impairment (MCI, a prodromal stage of AD). We conducted a meta-analysis to analyze the serum Mn levels in patients with AD and MCI. A systematic database search of PubMed, Web of Science, and the China National Knowledge Infrastructure (CNKI) identified 17 studies, including 836 cases and 1254 health controls (HC). Random-effects meta-analysis showed that patients with AD had significantly reduced serum Mn levels compared with HC subjects (SMD = −0.39; 95% CI (−0.71, −0.08); *p* = 0.015). MCI individuals had a tendency toward reduced serum Mn levels compared with HC subjects (SMD = −0.31; 95% CI (−0.70, 0.08); *p* = 0.117). A significant decrease in serum Mn levels was found in patients with cognitive impairment (including both AD patients and MCI patients) (SMD = −0.37, 95% CI (−0.60; −0.13); *p* = 0.002). Finally, no significant differences were observed between AD and MCI patients in serum levels (SMD = 0.24; 95% CI (−0.23, 0.72); *p* = 0.310). Our findings show that the serum Mn levels are lower in AD patients, and Mn deficiency may be a risk factor for AD.

## 1. Introduction

Alzheimer’s disease (AD) is a progressive neurodegenerative disorder clinically characterized by cognitive impairment, and becomes the predominant form of dementia [1,2,3]. Cognitive impairment commonly starts with mild symptoms and gradually aggravates [4]. Because of the slowness of the disease’s progression, the neurodegenerative processes are likely to start many years before AD patients presents with typical clinical symptoms of dementia. This transitional stage is clinically recognized as mild cognitive impairment (MCI), the precursor of dementia [5,6,7]. Despite great progress in basic and clinical studies of AD, the etiology of the disease is still largely unclear. Current treatments only offer symptomatic improvement without stopping disease progression [8]. Therefore, identifying the risk factors for dementia is important for effectively preventing or postponing the onset of AD [9,10].

It was found that the altered homeostasis of some metal elements could be related to the progression of AD [11], and the previous meta-analysis studies have indicated that AD is associated with an imbalance of increased Cu levels [12,13] and decreased Zn levels [12,14]. Manganese (Mn) widely exists in minerals, soil and food, and is an essential trace element for human health [15,16]. In the nervous system, Mn presents in several proteins and key enzymes, such as astrocytic glutamine synthetase, pyruvate carboxylase and mitochondrial superoxide dismutase [17,18,19,20], and is associated with some neurodegenerative disorders of the central nervous system (CNS) [21,22,23,24]. Recently, increasing evidence has shown that Mn is potentially involved in the progression of AD. It has been reported that AD patients have a deregulated metabolism of Mn, and a dysfunction of the manganese-superoxide dismutase (Mn-SOD) scavenger system, associated with the formation of senile plaques [25]. Reduced mitochondrial Mn-SOD activities have been found in the brain of neuropathology confirmed AD patients [26]. Moreover, it has been reported that the transport of Mn across the blood-brain barrier (BBB) is regulated by iron, and perturbed iron distribution has been implicated in the pathogenesis of AD [27,28,29]. 

Several studies have evaluated the associations between serum Mn levels and the risk of AD or MCI. However, conflicting results exist regarding whether cognitive impairment is associated with serum Mn levels. In addition, many studies have a relatively small sample size, which may not be sufficiently powered to detect the differences. Here, we performed a meta-analysis to study the association of serum Mn levels with AD and MCI.

## 2. Materials and Methods 

### 2.1. Search Strategy and Study Selection

This meta-analysis was conducted according to the Preferred Reporting Items for Systematic reviews and Meta-Analyses (PRISMA) statement [30]. The study protocol was registered with the International Prospective Register of Systematic Reviews (PROSPERO) (registration No. CRD42017055425). Supplementary materials showed the PRISMA Checklist. We searched published studies from the following databases: PubMed, Web of Science, and the China National Knowledge Infrastructure (CNKI) from inception to January 2017 reporting the association of serum Mn levels with AD or MCI. The keywords in the English or Chinese language included the following terms: Alzheimer’s disease, mild cognitive impairment, manganese, and serum. The search strategies are shown in Table S1. Eligible articles were retrieved from the above databases, and additional articles were obtained by handsearching the references of relevant studies. Studies for inclusion in this study should meet the following criteria: (1) a clinical study; (2) a case-control study; and (3) studies that provided a sample size and serum Mn levels in at least two groups of subjects (AD, MCI and HC). Exclusion criteria included: (1) in vitro or laboratory studies; (2) overlapped studies; (3) review or case reports; and (4) studies without serum Mn levels.

### 2.2. Data Extraction and Quality Assessment

Two investigators (Ke Du and Xin Zhong) independently assessed the eligible studies and extracted the relevant information from the literature, including the last name of first author, year of publication, geographic locations of studied populations, sample size, mean age of the subjects, percentage of women, criteria for AD diagnosis, and the technique used for measuring serum Mn levels. The serum Mn levels were expressed as the mean ± standard deviations (SD) if available, or estimated data from the sample size, median and range if they were not given directly [14,31]. The study quality was assessed using the Newcastle-Ottawa quality assessment Scale (NOS), in which scores for low (0–3), moderate (4–6), and high-quality studies (7–9) were assigned (Table S2). 

Meta-analyses were performed using STATA 12.0 (Stata, College Station, TX, USA). A random effects model was used to combine results from multiple studies if the heterogeneity was significant, or a fixed effects mode was used if the heterogeneity was not significant. Standardized Mean Difference (SMD), which expresses the difference in mean for the individual study, was used as the summary statistic. The heterogeneity among studies was evaluated using Chi-square and I-square tests. A subgroup analysis was performed to assess the impact of the study characteristics as possible sources of heterogeneity, including the methods for measuring Mn concentrations (ICP-MS (inductively coupled plasma-mass spectrometry), ICP-AES (coupled plasma-atomic emission spectrometry) or AAS (atomic absorption spectrometry) and the geographic locations of studied participants (Europe, Asia or Australia). Meta-regression was conducted to explore the effect of the continuous variables on the outcomes of the meta-analysis, especially the effect of two study-level characteristics (mean age and gender distribution) on the serum Mn levels in AD and MCI. A sensitivity analysis was performed to assess the influence of individual studies on the pooled SMD. Publication bias was assessed using the Egger’s and Begg’s tests. Cumulative meta-analysis was conducted to evaluate the temporal effect. The results were presented as forest plots and determined to be statistically significant when *p*-values were less than 0.05.

## 3. Results

### 3.1. Literature Search and Study Characteristics

**Figure 1:**
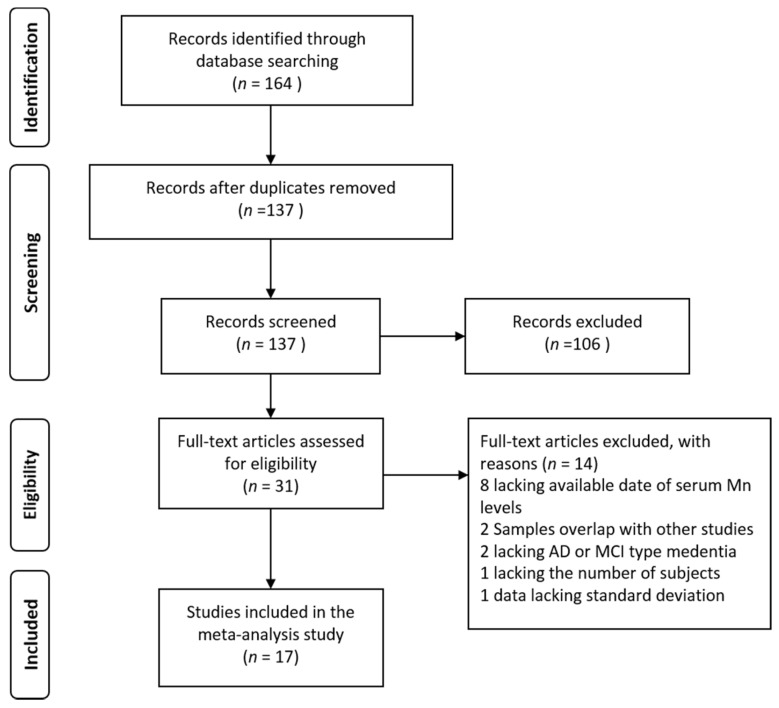
Flow diagram of the study selection process.

A total of 31 potential articles were found in an initial search using PubMed, Web of Science, and CNKI. Fourteen studies were excluded due to unavailability of serum Mn levels (*n =* 8), overlapped studies (*n =* 2), no AD or MCI (*n =* 2), insufficient subjects (*n =* 1), and no standard deviation (*n =* 1). Finally, 17 studies were included in this analysis (total 836 cases and 1254 controls). The selecting process was shown in a flow diagram (Figure 1). 

The sample size of the included studies ranged from 8 to 758. The average age of the patient groups ranged from 66.2 to 87.0 years. The proportion of female patients ranged from 33% to 80%. The geographic locations were in Europe, Asia, and Australia in 10, 4, and 3 studies, respectively. The average age was missing in one study, and the criteria for AD diagnosis was lacking in one study. The detailed characteristics are summarized in Table 1. The details of quality assessment scale according to the NOS are presented in Table S2. The study quality ranged from 7 stars (5 articles) to 8 stars (6 articles).

### 3.2. Studies on Mn Levels between Patients with AD and HC

**Figure 2:**
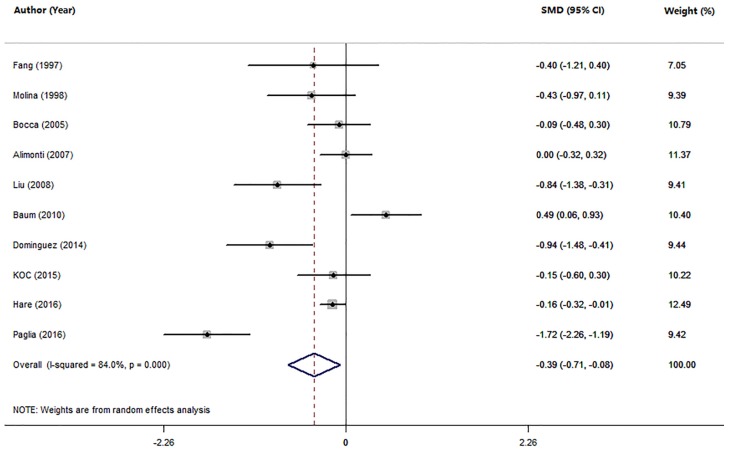
Forest plot for serum Mn levels in AD patients and health controls in included studies. The rhombus represents the combined effect estimates. The size of grey box is positively proportional to the weight assigned to each study, and horizontal lines represent the 95% confidence interval (CI).

Ten studies compared the serum Mn levels in AD patients with HC subjects (Table 1). The pooled sample size consisted of 1685 participants: 551 AD and 1134 HC. The random-effects meta-analysis results showed that patients with AD had significantly lower serum Mn levels than HC subjects (SMD = −0.39; 95% CI (−0.71, −0.08); *p* = 0.015; Figure 2). There was statistically significant heterogeneity among these studies (*I*^2^ = 84.0%, *p =* 0.000). The subgroup analysis assessment of the method for measuring serum Mn levels and geographic locations showed that the heterogeneity existed among studies, suggesting that the method for measuring Mn levels and geographic locations were not significant sources of heterogeneity (Table 2). In meta-regression analyses, neither mean age nor gender of AD patients were found to have moderating effects on the serum Mn levels in AD (mean age: *p =* 0.619; gender: *p =* 0.505). A sensitivity analysis showed that no study from the pooled analysis changed the results significantly. Temporal effect was excluded by using a cumulative analysis. Furthermore, there was no publication bias in the present meta-analysis evaluated by the Egger’s test (*p =* 0.258) and Begg’s test (*p =* 0.107). 

### 3.3. Studies on Mn Levels between Patients with MCI and HC

**Figure 3:**
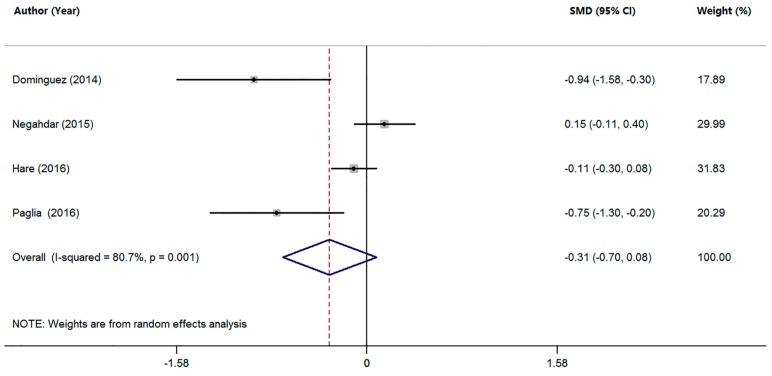
Forest plot for serum Mn levels in patients with MCI and health controls in included studies. The rhombus represents the combined effect estimates. The size of grey box is positively proportional to the weight assigned to each study, and horizontal lines represent the 95% confidence interval (CI).

Four studies compared the serum Mn levels in MCI patients with HC subjects (Table 1). The pooled sample size of these studies was 1233 participants, including 285 MCI patients and 948 HC subjects. The random-effects meta-analysis showed that MCI patients had a tendency toward decreased serum Mn levels compared with HC subjects, but no statistically significant difference was found (SMD = −0.31; 95% CI (−0.70, 0.08); *p =* 0.117; Figure 3). In addition, there was significant heterogeneity among these studies (*I*^2^ = 80.7%, *p =* 0.001). Due to the limited number of studies, no further analysis was performed.

### 3.4. Studies on Mn Levels between Cognitive Impairment Individuals and HC

**Figure 4:**
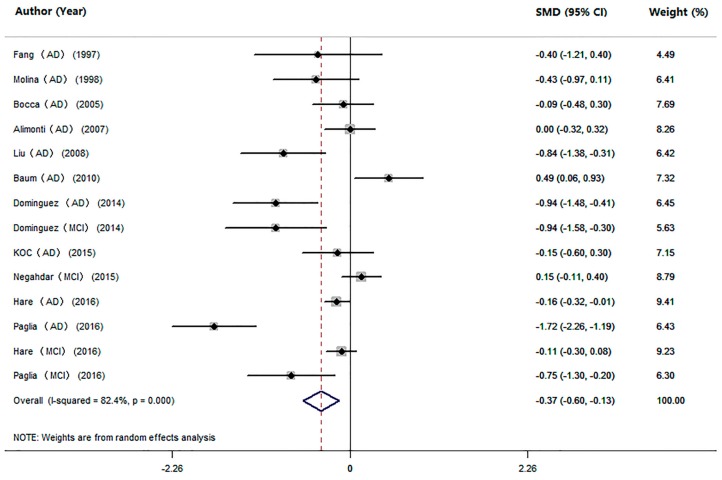
Forest plot for serum Mn levels in patients with cognitive impairment and health controls in included studies. The rhombus represents the combined effect estimates. The size of grey box is positively proportional to the weight assigned to each study, and horizontal lines represent the 95% confidence interval (CI).

We also performed an analysis of the difference in serum Mn levels between cognitive impairment individuals (AD and MCI pooled together) and HC subjects in 14 studies (Table 1). The pooled sample size consisted of 2090 participants, including 836 cognitive impairment individuals and 1254 HC subjects. The meta-analysis results showed that patients with cognitive impairment had significantly lower serum Mn levels compared with HC subjects (SMD = −0.37, 95% CI (−0.60; −0.13); *p* = 0.002; Figure 4). Significant heterogeneity (*I*^2^ = 82.4%, *p =* 0.000) was observed across the studies. To explore the possible source of heterogeneity, a subgroup analysis was conducted, and the heterogeneity was not removed by the method for measuring Mn levels or geographic locations of the studied population (Table 3). Since the proportion of female patients ranged from 33% to 80% (Table 1), which suggested high heterogeneity, we repeated the analysis after excluding the studies that had female proportions of the maximum and the minimum (Paglia MCI 2016; Fang AD 1997). The results also showed lower levels of Mn in patients with cognitive impairment than in HC subjects (SMD = −0.34; 95% CI (−0.58, −0.09); *p =* 0.007), indicating good stability of our meta-analysis. Further, meta-regression analyses showed that mean age and gender were not the sources of heterogeneity (mean age: *p =* 0.771; gender: *p =* 0.636). Sensitivity analyses showed that no studies significantly changed the overall results. No temporal effect was found by the cumulative meta-analysis. Furthermore, according to the Egger’s (*p =* 0.068) or Begg’s (*p =* 0.063) tests, no publication bias was observed in the meta-analysis.

### 3.5. Studies on Mn Levels between Individuals with AD and MCI

**Figure 5:**
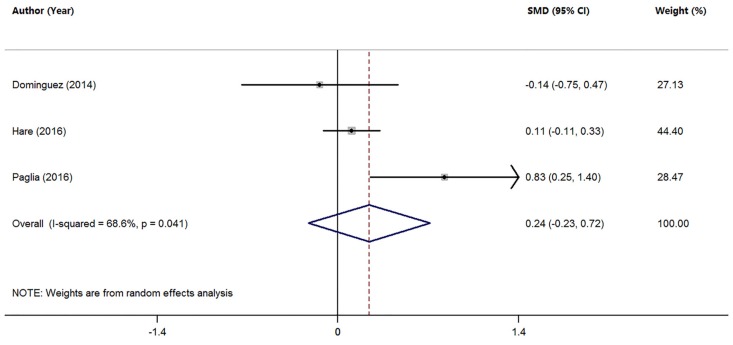
Forest plot for serum Mn levels in patients with AD and MCI in included studies. The rhombus represents the combined effect estimates. The size of grey box is positively proportional to the weight assigned to each study, and horizontal lines represent the 95% confidence interval (CI).

Three studies analyzed the differences in serum Mn levels between AD and MCI patients (Table 1). The pooled sample size was 435 subjects, including 165 MCI patients and 270 AD patients. The random-effects meta-analysis showed that MCI patients had similar serum Mn levels compared with AD patients (SMD = 0.24; 95% CI (−0.23, 0.72); *p =* 0.310; Figure 5). There was significant heterogeneity among these studies (*I*^2^ = 68.6%, *p =* 0.041). Since the meta-analysis included a limited number of studies, and found no difference in serum Mn levels between MCI and AD patients, no further test was conducted. 

## 4. Discussion

To date, the association of serum Mn levels with cognitive impairment remains controversial. Some studies have shown that serum Mn levels are reduced in AD patients or MCI patients compared with HC subjects [11,32,36,38,39]. However, several other studies have reported that AD and MCI patients have similar or higher serum Mn levels compared with HC subjects [33,34,35,37,40,41]. In this meta-analysis, we investigated the association of serum Mn levels with AD and MCI. We found that AD patients had significantly lower serum Mn levels compared with HC subjects (SMD = −0.39; 95% CI (−0.71, −0.08); *p =* 0.015), and MCI patients tended to have lower serum Mn levels (SMD = −0.31; 95% CI (−0.70, 0.08); *p =* 0.117). A decrease in serum Mn levels was found in patients with cognitive impairment including both AD patients and MCI patients. However, strong heterogeneity existed among the studies. Heterogeneity was not due to methods for measuring Mn levels, geographic locations, age, and gender of patients. In this meta-analysis, we found that the serum Mn levels were significantly lower in AD patients compared with HC subjects. Although we found that MCI patients had a tendency toward a decrease in the serum Mn levels, no statistical significance was found. The smaller number of studies and sample size (4 studies, 285 MCI subjects and 948 health controls) (compared with 10 studies, 551 AD patients and 1134 health controls in AD studies) may contribute to the no statistically significant difference between the serum Mn levels in MCI patients and HC subjects. In two meta-analysis studies [11,39], the serum Mn levels were found to be significantly decreased in MCI patients compared with health controls, but in two other studies [40,41] Mn levels were not significantly different between MCI and HC subjects. Hence, the conclusion was not robust and further investigations are necessary to address serum Mn levels in MCI individuals. 

Consistent with our results showing that AD patients had lower serum Mn levels, Szabo et al. [42] found that the Mn levels were lower in the frontal cortex tissues of AD patients. In addition, Gerhardsson et al. [43] reported that the Mn levels in cerebrospinal fluid was significantly lower in AD patients. The mechanisms underlying lower Mn levels in the brain of AD patients remain unknown. Multiple transporters, such as the transferrin receptor, the divalent metal transporter 1 and the dopamine transporter, have been found to regulate Mn levels to maintain Mn homeostasis in the brain [15]. Dysfunction of these Mn transporters has been found in AD patients or in AβPP/PS1 transgenic AD mice [44,45,46]. Therefore, the low Mn levels in the brain of AD patients may be the result dysfunctional Mn transporters. However, we cannot rule out the possibility that dietary Mn deficiency is involved in Mn decrement in AD. Further studies will be required to clarify the molecular mechanisms that are responsible for Mn deficiency in AD patients.

It remains unclear how Mn reduction contributes to AD progression. It is known that Mn is important for several key enzymes, such as glutamine synthetase, arginase, pyruvate carboxylase, and Mn-SOD. These metalloproteins regulate several enzymatic processes, including antioxidant defense, energy metabolism and immune function, and dysfunction of these metalloproteins contribute to the pathogenesis of AD [15,47,48,49,50]. Mn deficiency may promote the progression of AD through these metalloproteins. Our findings that AD patients had lower serum Mn levels support the notion that Mn deficiency is a potential risk factor for AD, and Mn related intervention is a potential therapy for the prevention of AD. Although the results in this study were not completely indicative of causation, such evidence may indicate a role for low Mn in the degenerative conditions of AD. Our meta-analysis still has some limitations. First, the number of studies are relatively small, especially for the studies in MCI subjects. Future studies with larger sample sizes are required to confirm our conclusion. Second, Mn, as one of most important micronutrients for human health, naturally exists in daily diets [51,52]. Thus, the serum Mn levels are affected by dietary intake of Mn. Due to the unavailability of the dietary intake of Mn in the included studies, we could not assess the possible associations between dietary intake of Mn and serum Mn levels. Third, since the methods used for measuring serum Mn and the sampling techniques are different among studies, the data of the mean serum Mn levels exhibited obvious variability among the included studies. Fourth, the studies published in English or Chinese have been reviewed using our research database, but we excluded the studies published in other languages.

In summary, this meta-analysis found that there were significantly lower serum Mn levels in patients with cognitive impairment (AD and MCI patients) compared with HC subjects. However, the results should be interpreted with caution due to the high heterogeneity of the studies.

## Figures and Tables

**Table 1:** Characteristics of the included studies in the meta-analysis of serum Mn levels.

**Studies on AD to HC**											
		**AD Patients**					**HC Subjects**				
**Reference**	**Country**	***n***	**Gender**	**Age**	**Mn Concentration**	**Criteria for AD Diagnosis**	***n***	**Gender**	**Age**	**Mn Concentration**	**Method**
			**(% Female)**	**Mean ± SD (Year)**	**Mean ± SD (µg/L)**			**(% Female)**	**Mean ± SD (Year)**	**Mean ± SD (µg/L)**	
Fang 1997 [32]	China	24	33	61–87	42.85 + 17.03	DSM-III	8	38	58–72	50.00 + 19.78	ICP-AES
Molina 1998 [33]	Spain	26	46	73.1 ± 8.2	1.03 + 0.68	DSM-IV, NINCDS-ADRDA criteria	28	43	70.8 ± 7.3	1.31 + 0.63	AAS
Bocca 2005 [34]	Italy	60	67	74.6 ± 6.4	0.63 + 0.22	NINCDS-ADRDA criteria	44	25	≥45	0.65 + 0.24	ICP-MS
Alimonti 2007 [35]	Italy	53	68	74.5 ± 6.5	0.60 + 0.08	NINCDS-ADRDA criteria	124	35	44.8 ± 12.7	0.60 + 0.04	ICP-MS
Liu 2008 [36]	China	30	47	66.2 ± 9.9	15.00 ± 4.00	DSM-IV, NINCDS-ADRDA criteria	28	46	66.8 ± 8.3	18.00 ± 3.00	ICP-AES
Baum 2010 [37]	Hong Kong	44	66	74.3 ± 8.7	1.18 ± 1.15	NINCDS-ADRDA criteria	41	49	79.1 ± 6.0	0.73 ± 0.51	ICP-MS
Dominguez 2014 [11]	Spain	30	60	80.9 ± 4.5	0.62 ± 0.35	NINCDS-ADRDA criteria	30	57	74.0 ± 5.7	1.16 ± 0.73	ICP-MS
KOC 2015 [38]	Turkey	44	49	77.7 ± 9.3	9.00 ± 7.50	DSM-IV, NINCDS-ADRDA criteria	33	52	73.2 ± 10.6	10.00 ± 5.00	ICP-MS
Paglia 2016 [39]	Italy	34	74	72.4 ± 7.5	0.59 ± 0.32	NINCDS-ADRDA criteria	40	63	65.5 ± 6.4	1.24 ± 0.42	ICP-MS
Hare 2016 [40]	Australia	206	62	78.0 ± 8.6	0.82 ± 0.25	-	758	31	70.0 ± 7.0	0.92 ± 0.70	ICP-MS
**Studies on MCI to HC**											
		**MCI Individuals**					**HC Subjects**				
**Reference**	**Country**	***n***	**Gender**	**Age**	**Mn Concentration**	**Criteria for AD Diagnosis**	***n***	**Gender**	**Age**	**Mn Concentration**	**Method**
			**(% Female)**	**Mean ± SD (Year)**	**Mean ± SD (µg/L)**			**(% Female)**	**Mean ± SD (Year)**	**Mean ± SD (µg/L)**	
Dominguez 2014 [11]	Spain	16	38	75.9 ± 5.7	0.57 ± 0.33	-	30	57	74.0 ± 5.7	1.16 ± 0.73	ICP-MS
Negahdar 2015 [41]	Iran	120	50	74.3 ± 7.8	14.30 + 5.18	-	120	50	67.7 ± 6.9	13.50 + 5.30	AAS
Paglia 2016 [39]	Italy	20	80	68.3 ± 7.8	0.91 ± 0.48	-	40	63	65.5 ± 6.4	1.24 ± 0.42	ICP-MS
Hare 2016 [40]	Australia	129	57	75.7 ± 7.6	0.85 ± 0.37	-	758	31	70.0 ± 7.0	0.92 ± 0.70	ICP-MS
**Studies on MCI to AD**											
		**AD patients**					**MCI Individuals**				
**Reference**	**Country**	***n***	**Gender**	**Age**	**Mn Concentration**	**Criteria for AD Diagnosis**	***n***	**Gender**	**Age**	**Mn Concentration**	**Method**
			**(% Female)**	**Mean ± SD (Year)**	**Mean ± SD (µg/L)**			**(% Female)**	**Mean ± SD (Year)**	**Mean ± SD (µg/L)**	
Dominguez 2014 [11]	Spain	30	60	80.9 ± 4.5	0.62 ± 0.35	NINCDS-ADRDA criteria	16	38	75.9 ± 5.7	0.57 ± 0.33	ICP-MS
Paglia 2016 [39]	Italy	34	74	72.4 ± 7.5	0.59 ± 0.32	NINCDS-ADRDA criteria	20	80	68.3 ± 7.8	0.91 ± 0.48	ICP-MS
Hare 2016 [40]	Australia	206	62	78.0 ± 8.6	0.82 ± 0.25	-	129	57	75.7 ± 7.6	0.85 ± 0.37	ICP-MS

NINCDS-ADRDA, National Institute of Neurological and Communicative Disorders and Stroke-Alzheimer’s Disease and Related Disorders Association; DSM-III or DSM-IV, the Diagnostic and Statistical Manual for Mental Disorders; ICP-MS, inductively coupled plasma-mass spectrometry; ICP-AES, inductively coupled plasma-atomic emission spectrometry; AAS, atomic absorption spectrometry; MCI, mild cognition impairment.

**Table 2:** Meta-analysis of studies on serum Mn levels between AD patients and health controls.

Subgroups	*n* of Studies	SMD (95% CI)	*I*^2^	*p*-Value
All studies	10	−0.39 (−0.71, −0.08)	84.0%	0.000
**Methods**				
ICP-MS	7	−0.33 (−0.73, 0.06)	88.1%	0.000
ICP-AES	2	−0.71 (−1.16, −0.26)	0.0%	0.373
AAS	1	−0.43 (−0.97, 0.11)	-	-
**Geographic locations**				
Europe	6	−0.53 (−1.03, −0.04)	86.4%	0.000
Asia	3	−0.23 (−1.14, 0.68)	86.8%	0.001
Australia	1	−0.16 (−0.32, −0.01)	-	-

**Table 3:** Meta-analysis of studies on serum Mn levels between patients with cognitive impairment and health controls.

Subgroups	*n* of Studies	SMD (95% CI)	*I*^2^	*p*-Value
All studies	14	−0.37 (−0.60, −0.13)	82.4%	0.000
**Methods**				
ICP-MS	10	−0.38 (−0.66, −0.10)	85.0%	0.000
ICP-AES	2	−0.71 (−1.16, −0.26)	0.0%	0.373
AAS	2	−0.09 (−0.64, 0.46)	71.8%	0.060
**Geographic locations**				
Europe	8	−0.60 (−1.01, −0.20)	82.8%	0.000
Asia	4	−0.11 (−0.65, 0.43)	81.7%	0.001
Australia	2	−0.14 (−0.26, −0.02)	0.0%	0.651

## Supplementary Materials

The following are available online at http://www.mdpi.com/2072-6643/9/3/231/s1, Table S1: Full search terms and search strategy used for systematically reviewing the studies, Table S2: Quality assessment according to the nine-star Newcastle-Ottawa Scale (NOS), Checklist S1: PRISMA checklist.
