# Supplementary material for: Association of Serum Manganese Levels with Alzheimer’s Disease and Mild Cognitive Impairment: A Systematic Review and Meta-Analysis
Source: Nutrients. 2017 Mar 3;9(3):231. doi: 10.3390/nu9030231 (PMC5372894; doi:10.3390/nu9030231)
Supplement: Supplementary file 1 [file nutrients-09-00231-s001.zip › nutrients-172646-supplementary/nutrients-172646-supplementary.docx]

Supplementary Materials: Association of Serum Manganese Levels with Alzheimer’s Disease and Mild Cognitive Impairment: A Meta-Analysis Study

Ke Du, Mingyan Liu, Yanzhu Pan, Xin Zhong and Minjie Wei

**Table S1.** Full search terms and search strategy used for systematically reviewing the studies.

| **No.** | **Search Terms** |
| --- | --- |
| #1 | “Alzheimer’s disease (Title/Abstract)  OR “mild cognitive impairment” (Title/Abstract) |
| #2 | “manganese” (Title/Abstract) |
| #3 | “serum” (Title/Abstract) |
| 4 | Combination 1 AND 2 AND 3 |

**Table S2.** Quality assessment according to the nine-star Newcastle-Ottawa Scale (NOS).

| **ID** | **Author** | **Year** | **Country** | **Selection** | | | | **Comparability** | | **Exposure** | | | **Overall Quality** |
| --- | --- | --- | --- | --- | --- | --- | --- | --- | --- | --- | --- | --- | --- |
|  |  |  |  | 1 | 2 | 3 | 4 | 5A | 5B | 6 | 7 | 8 |  |
| 1 | Fang [1] | 1997 | China | * | * | * | * | * |  | * | * | * | 8 |
| 2 | Molina [2] | 1998 | Spain | * | * |  | * | * | * | * | * | * | 8 |
| 3 | Bocca [3] | 2005 | Italy | * | * |  | * | * |  | * | * | * | 7 |
| 4 | Alimonti [4] | 2007 | Italy | * | * | * | * | * |  | * | * | * | 8 |
| 5 | Liu [5] | 2008 | China | * | * |  | * | * |  | * | * | * | 7 |
| 6 | Baum [6] | 2010 | Hong Kong | * | * |  | * | * |  | * | * | * | 7 |
| 7 | Dominguez [7] | 2014 | Spain | * | * |  | * | * |  | * | * | * | 7 |
| 8 | KOC [8] | 2015 | Turkey | * | * |  | * | * |  | * | * | * | 7 |
| 9 | Negahdar [9] | 2015 | Iran | * | * |  | * | * | * | * | * | * | 8 |
| 10 | Paglia [10] | 2016 | Italy | * | * |  | * | * | * | * | * | * | 8 |
| 11 | Hare [11] | 2016 | Australia | * | * |  | * | * | * | * | * | * | 8 |

A study can be awarded a maximum of one star for each numbered item within the Selection and Exposure categories and a maximum of two stars for Comparability. Scores for low (0–3), moderate (4–6), and high-quality studies (7–9) were assigned

References

1. Fang, B.; Zhao, G.; Jin, J.; Zhang, Y.; Hu, J.; Fang, S. Determination and discussion of seven trace elements in serum of the patients with senile dementia. *Acta Nutrimenta Sinica* **1997**, *19*, 88–90.
2. Molina, J.A.; Jimenez-Jimenez, F.J.; Aguilar, M.V.; Meseguer, I.; Mateos-Vega, C.J.; Gonzalez-Munoz, M.J.; de Bustos, F.; Porta, J.; Orti-Pareja, M.; Zurdo, M*.*; et al. Cerebrospinal fluid levels of transition metals in patients with alzheimer’s disease. *J. Neural. Transm.* **1998**, *105*, 479–488.
3. Bocca, B.; Forte, G.; Petrucci, F.; Pino, A.; Marchione, F.; Bomboi, G.; Senofonte, O.; Giubilei, F.; Alimonti, A. Monitoring of chemical elements and oxidative damage in patients affected by alzheimer’s disease. *Ann. Ist. Super. Sanita* **2005**, *41*, 197–203.
4. Alimonti, A.; Ristori, G.; Giubilei, F.; Stazi, M.A.; Pino, A.; Visconti, A.; Brescianini, S.; Sepe Monti, M.; Forte, G.; Stanzione, P.; et al. Serum chemical elements and oxidative status in alzheimer’s disease, parkinson disease and multiple sclerosis. *Neurotoxicology* **2007**, *28*, 450–456.
5. Liu, K. The study of the association between trace element and senile dementia/depressive disorder. Master’s Thesis, Shandong University, Shandong, China, 2008.
6. Baum, L.; Chan, I.H.; Cheung, S.K.; Goggins, W.B.; Mok, V.; Lam, L.; Leung, V.; Hui, E.; Ng, C.; Woo, J.; et al. Serum zinc is decreased in alzheimer’s disease and serum arsenic correlates positively with cognitive ability. *Biometals* **2010**, *23*, 173–179.
7. Gonzalez-Dominguez, R.; Garcia-Barrera, T.; Gomez-Ariza, J.L. Characterization of metal profiles in serum during the progression of alzheimer’s disease. *Metallomics* **2014**, *6*, 292–300.
8. Koc, E.R.; Ilhan, A.; Zubeyde, A.; Acar, B.; Gurler, M.; Altuntas, A.; Karapirli, M.; Bodur, A.S. A comparison of hair and serum trace elements in patients with alzheimer disease and healthy participants. *Turk. J. Med. Sci.* **2015**, *45*, 1034–1039.
9. Negahdar, H.; Hosseini, S.R.; Parsian, H.; Kheirkhah, F.; Mosapour, A.; Khafri, S.; Haghighi, A.H. Homocysteine, trace elements and oxidant/antioxidant status in mild cognitively impaired elderly persons: A cross-sectional study. *Rom. J. Intern. Med.* **2015**, *53*, 336–342.
10. Paglia, G.; Miedico, O.; Cristofano, A.; Vitale, M.; Angiolillo, A.; Chiaravalle, A.E.; Corso, G.; Di Costanzo, A. Distinctive pattern of serum elements during the progression of alzheimer’s disease. *Sci. Rep.* **2016**, *6*, 22769.
11. Hare, D.J.; Faux, N.G.; Roberts, B.R.; Volitakis, I.; Martins, R.N.; Bush, A.I. Lead and manganese levels in serum and erythrocytes in alzheimer’s disease and mild cognitive impairment: Results from the australian imaging, biomarkers and lifestyle flagship study of ageing. *Metallomics* **2016**, *8*, 628–632.
